# The pillars of health: influence of multiple lifestyle behaviors on body mass index and depressive symptoms in adult twins

**DOI:** 10.1186/s12889-022-13901-7

**Published:** 2022-08-05

**Authors:** Glen E. Duncan, Ally R. Avery, Siny Tsang, Nathaniel F. Watson, Bethany D. Williams, Eric Turkheimer

**Affiliations:** 1 Department of Nutrition and Exercise Physiology, Elson S. Floyd College of Medicine, Washington State University Health Sciences Spokane, 412 E. Spokane Falls Blvd, Spokane, USA; 2 Department of Neurology, University of Washington School of Medicine, 1959 NE Pacific St, Seattle, WA 98195 USA; 3 University of Washington Medicine Sleep Center, 908 Jefferson St, Seattle, WA 98104 USA; 4 Department of Psychology, University of Virginia, 485 McCormick Road, Gilmer Hall, Room 102, Charlottesville, VA 22903 USA

**Keywords:** Fruits and Vegetables, Lifestyle Behaviors, Physical Activity, Prevention, Sedentary Behaviors, Sleep, Smoking, Twins

## Abstract

**Background:**

Guidelines promoting healthy lifestyles are cornerstones of chronic disease prevention and treatment. The purpose of this study is to investigate independent and joint associations of five key health behaviors with health outcomes (body mass index (BMI kg/m^2^) and depressive symptoms) in adult twins.

**Methods:**

We included 6,048 twin pairs from a community-based registry. Five key health behaviors were: (1) ≥ 8 h of sleep per night, (2) ≥ 5 servings of fruits and vegetables daily, (3) ≤ 2 h sedentary time per day, (4) ≥ 150 min of moderate-to-vigorous physical activity (MVPA) per week, and (5) no smoking. We analyzed phenotypic associations between behaviors and outcomes; whether phenotypic associations were confounded by additive genetic and shared environmental factors within twin pairs (“quasi-causal” associations); and which behaviors, considered simultaneously, had the largest associations with outcomes.

**Results:**

We found negative phenotypic associations between number of behaviors achieved with BMI and depressive symptoms score (*ps* < 0.05). Associations remained significant, though attenuated, when controlling for genetic and shared environmental factors, and demographics, for depressive symptoms score but not BMI (*p* < 0.05). Quantitative variable importance measures derived from regression tree models showed sedentary time and MVPA were the most important variables in partitioning twins with different BMI, and smoking and sedentary time for partitioning twins with different depressive symptoms score.

**Conclusions:**

Achievement of commonly endorsed health behaviors is associated with lower BMI (especially sedentary and MVPA targets) and depressive symptoms score (especially sedentary and smoking targets). This provides further support of health behavior promotion to improve health outcomes.

**Supplementary Information:**

The online version contains supplementary material available at 10.1186/s12889-022-13901-7.

## Introduction

Guidelines promulgated by government and scientific agencies to promote healthy lifestyle behaviors are cornerstones of prevention and treatment efforts for numerous chronic diseases. Frequently advocated modifiable health behaviors include: sleeping at least 7 h per night [1], consuming 5 or more servings per day of fruits and vegetables [2, 3], limiting time in sedentary behaviors [4, 5], obtaining at least 150 min per week of moderate-to-vigorous physical activity (MVPA) [5–7], and not smoking [8]. Despite myriad evidence that following these guidelines are associated with reductions in chronic disease, morbidity, and mortality [1–4, 8], many US adults fail to meet various behavioral targets alone or in combination [9–12]. Further, undesirable health outcomes related to these health behaviors, such as obesity [13] and depression [14], are prevalent in the US. This is cause for public health concern because those diagnosed with obesity and/or depression are at higher risk of experiencing reduced quality of life, social stigmatization, and early mortality [13, 14]. Further, cardiovascular disease and depression are the first and second leading causes of disability, respectively, across the globe. [15]. Use of epidemiology and surveillance of behavioral health risk factors is necessary to fully understand how achievement of key health behavior guidelines is related to disease, and how to effectively promote health nationally.

Interventions to improve multiple health behaviors have been conducted with moderate efficacy to improve health outcomes [16]. Given the continued low prevalence of meeting behavioral recommendations across the US population, there is a need for research to inform how success of achieving health behavior changes may be associated with improved health [17]. This study used twin pairs from a community-based twin registry to accomplish two primary aims. First, to examine the association between achievement of five key health behaviors (i.e., “pillars”) and health outcomes highly prevalent in the US (high body mass index (BMI kg/m^2^) and depressive symptoms). The twin study design allows us to control for genetic and shared environmental confounds when estimating the associations between health behaviors and health outcomes, to provide a more robust investigation of the relationship than traditional correlation analyses. We hypothesized that achievement of all five pillars would be associated with significantly lower BMI and lower depressive symptoms, both between and within twins. Second, we explored which health behavior pillars, when considered simultaneously, are the most important predictors of the health outcomes of interest. Such analyses were conducted to investigate whether meeting certain combinations of health pillars, rather than all five pillars, could also be related to improved health, for the purpose of improving efficacy of behavior change efforts. We hypothesized that achievement of certain combinations of health behavior pillars is associated with significantly lower BMI and lower depressive symptoms, both between and within twins.

## Methods

### Participants

This study included a sample of 6,048 twin pairs from the community-based Washington State Twin Registry and used a cross-sectional study design. Twins included monozygotic (MZ) and dizygotic (DZ) male and female twin pairs of the same sex, aged 18–97 years, reared together. Participants were recruited from Washington State driver’s license and identification card applications; details on recruitment are described in detail elsewhere [18–20]. All twins completed an enrollment survey with five questions addressing childhood similarity to determine zygosity (MZ vs. DZ), a common twin registry practice with an accuracy of 95–98% compared to biological indicators [21–23]. Data collected from completed surveys between 2008–2018 were analyzed. All members of the Registry provide written informed consent to participate in research.

### Measures

#### Health Outcomes

The primary outcome of interest, BMI, was calculated from self-reported height and weight and expressed as kg/m^2^. The height and weight measures were collected from responses to the survey questions “What is your current height?” in feet and inches and “What is your current weight?” in pounds. We found excellent agreement between self-reported and measured BMI (26.7 vs. 27.2 kg/m^2^, respectively; *r* = 0.97) among a sample of 1,113 individual twin participants across five in-person studies, suggesting the use of self-reported height and weight for BMI is a robust measure.

The secondary outcome of interest, participant’s self-reported level of depressive symptoms, was assessed using the 2-item Patient Health Questionnaire-2 (PHQ-2) [24]. Participants responded to the frequency of depressed mood and anhedonia over the last two weeks on a 4-point Likert-type scale (0 = *Not at all*, 1 = *Several days*, 2 = *More than half the days,* 3 = *Nearly every day*). A total depressive symptoms score was obtained by summing the two items, with higher scores (maximum = 6) reflecting higher levels of depressive symptoms.

#### Health “*Pillars*”

The five health behavior pillars of interest were: (1) ≥ 8 h of sleep per night, (2) ≥ 5 servings of fruits and vegetables daily, (3) ≤ 2 h of sedentary time daily, (4) ≥ 150 min of moderate-to-vigorous physical activity (MVPA) per week, and (5) no smoking. These criteria were assessed using the self-report questionnaires when the twins enrolled in the WSTR; the relevant survey questions are described below.

Sleep was assessed using self-report of the amount of total sleep per night recorded in hours and minutes (*On average, how long do you sleep per night* [25]*?*); those reporting at least 8 h of sleep met this criterion in the main analysis, whereas those reporting at least 7 h of sleep met this criterion in sensitivity analyses. The rationale for this selection is based on panel consensus where 7–9 h of sleep were deemed appropriate to support optimal health in adults (i.e., 8 h standard is the mid-point of the range) and 7 h represents the lower end of the range [1]. Fruits and vegetables were assessed separately using a single question: *During the past 4 weeks, how many servings of the following did you eat on a typical day?* Participants who reported having (i) 5 or more servings of fruits and at least 1 to 2 servings of vegetables, (ii) 3 to 4 servings of fruits and at least 1 to 2 servings of vegetables, (iii) 5 servings of vegetables and at least 1 to 2 servings of fruits, or (iv) 3 to 4 servings of fruits and at least 1 to 2 servings of vegetables were coded as meeting this criterion. Sedentary time was assessed using the question: *Over the past 4 weeks, how much time altogether did you spend on a typical day sitting and watching TV or videos or using a computer outside of work?* Participants who reported 0 h or 1–2 h were coded as meeting this criterion, whereas those who reported 3–4 h or 5 or more hours were coded as not meeting this criterion. Physical activity was assessed through twins reporting the number of days per week they engaged in vigorous physical activity for at least 20 min and moderate physical activity for at least 30 min. The total minutes per week in MVPA was computed by summing moderate and vigorous physical activity days by their respective durations. The MVPA measure provides an estimate that directly corresponds to activity levels recommended for health; those who met at least 150 min of MVPA per week were coded as meeting this criterion [6, 7]. In a sample of 277 individual twins who wore accelerometers over two weeks of monitoring, the correlation between objective and subjective measures of MVPA was *r* = 0.48 (95% CI = 0.39 – 0.57, *p* < 0.001). Smoking was also assessed through self-report; participants who reported as not currently smoking were coded as meeting this criterion.

Each of five health pillars was coded as a dichotomous variable, with 0 (No) indicating not meeting the criterion, and 1 (Yes) indicating meeting the criterion. A sixth predictor variable, the number of health pillars, was obtained by summing the number of health pillars met in any combination. The number of health pillars met ranged from 0 (*did not meet any of the five health pillars*) to 5 (*met all five health pillars*).

#### Covariates

Age, sex, race, annual household income, and education level collected from the enrollment survey questions were used as covariates in the statistical analyses. Age was calculated based on the reported date of birth. Sex was self-reported as male or female. Race was reported using six response options (American Indian or Alaska Native, Black or African American, Native Hawaiian or Pacific Islander, Asian, White, and Other), which was subsequently re-categorized into White and non-White. Annual household income was self-reported in eight categories, ranging from “less than $20,000” to “$80,000 or more.” Education referred to the highest level of education completed: less than high school, high school graduate/GED, some college, bachelor’s degree, and graduate/professional degree.

### Statistical analysis

Height and weight data to calculate BMI were missing for 143 participants (1.2%), depressive symptoms score was missing for 138 participants (1.1%), and number of health pillars met was missing for 463 (3.8%). These observations were omitted from the descriptive statistics; they were included in the structural equation modeling analyses using full information maximum likelihood (FIML) to account for missingness. Descriptive statistics for participants were computed and reported for the overall sample and stratified by sex.

#### Twin model

We used the classical twin model to decompose the variances of BMI, depressive symptoms score, and number of health behaviors into three components: additive genetic (A), shared environmental (C), and non-shared environmental (E) factors. Details of this *univariate twin model* are described in the Additional Files (pg. 2) and illustrated in Fig. A1. We next utilized the twin design to examine the relation between the endorsement of each health pillar and health outcomes. Details of this *bivariate twin model* are also described in the Additional Files (pgs. 2–3) and illustrated in Fig. A2.

**Fig. 1:**
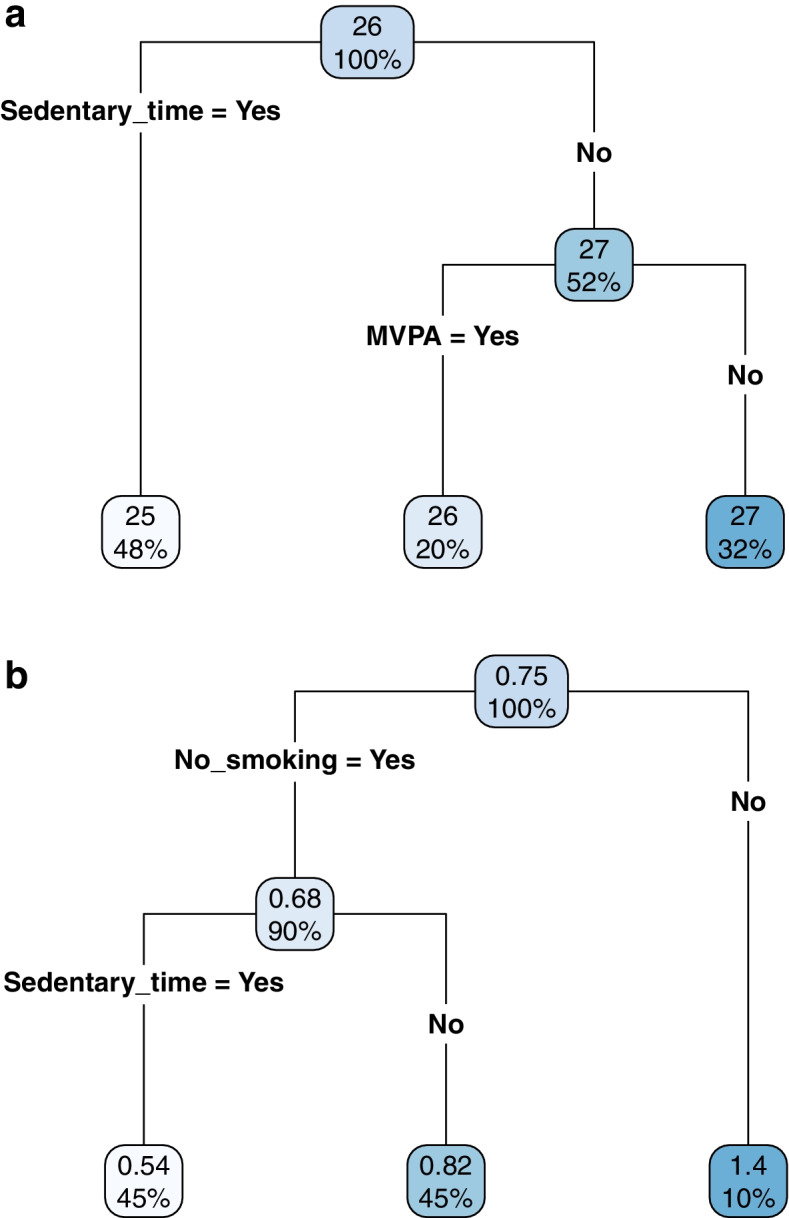
**a** Regression tree model predicting BMI with the five health pillars. **b** Regression tree model predicting depressive symptoms (PHQ-2 score) with the five health pillars. The numbers in the node represent the average BMI/PHQ-2 score among the corresponding subgroup of participants. The percentages in the node represent the proportion of participants in the corresponding subgroup

**Fig. 2:**
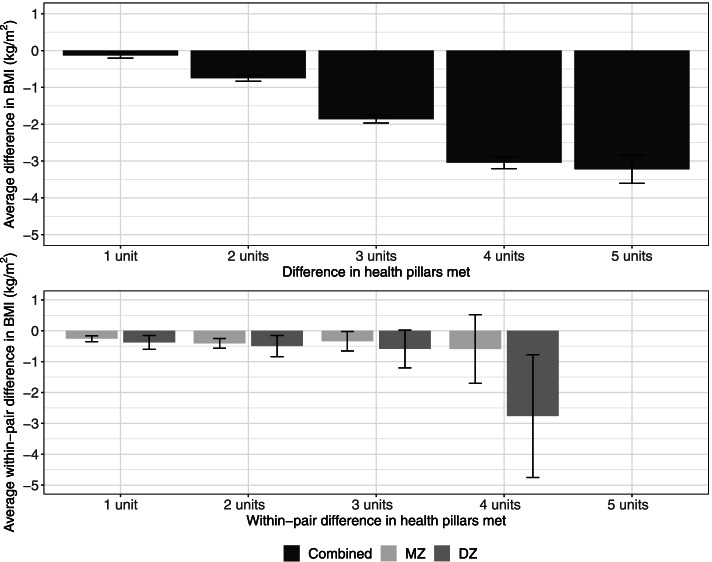
Average difference in BMI across individuals meeting different number of health pillars (top panel) and average within-pair difference in BMI between twin pairs meeting different number of health pillars by zygosity (bottom panel). Error bars denote standard errors

Body mass index (BMI) and depressive symptoms were expressed as continuous variables in all statistical analyses. As BMI is not normally distributed, we used its natural logarithm for analyses. Because the distribution of PHQ-2 is positively skewed, the depressive symptoms scores were square root transformed for these analyses. Each health pillar was modeled using a categorical variable model that posits a normally distributed latent continuous liability to the endorsement of health pillar; latent cutoffs on the distribution determine placement of participants in non-endorsement versus endorsement of the corresponding health pillar [26].

#### Regression tree model

We used regression tree models to explore which of the five health pillars, when considered simultaneously, were the most important predictors of health outcomes (BMI and depressive symptoms score). Regression tree models are non-parametric methods that recursively partition the data into increasingly homogenous subgroups until no further improvements can be made. Regression tree models are useful as they make no prior assumptions regarding the distribution of the predictor variables and are able to examine higher-order interactions among predictors before identifying variables to be included in the model [27, 28].

The regression tree models were conducted using the “rpart” package [29] in R, implementing the classification and regression tree (i.e., CART [30]) method. Gini index, a measure of subgroup variability [31], is used to determine the choice of splits. The predictor variable that provides the greatest reduction in Gini index is used for the next split [28]. The process is subsequently applied to each subgroup and continues recursively until no further improvements can be made. In cases of missing data, surrogate splits are used in which the variable that can achieve the next best split is used to partition the data. All regression tree models were derived using tenfold cross-validation, with 20 minimum cases in the parent node. Based on the regression tree models, a measure of variable importance is available [32]. It is computed as the sum of the improvement measure due to each predictor in each split for which it was a primary or candidate (i.e., important but not used in the actual split) splitter. The variable importance measure is scaled from 0 (the least important) to 100 (the most important).

Based on results from the regression tree models, we used the bivariate twin model (described in the Additional Files Methods) to investigate the association between the number of health pillars and health outcomes (BMI or depressive symptoms). Two sets of analyses (Models 1 to 3 as described in the Additional Files ) were performed for each health outcome (BMI and depressive symptoms), one for all five pillars (zero to a maximum of five pillars) and one for the most “important” pillars (zero to the number of pillars identified from the regression tree models). Number of health pillars was modeled using a categorical variable model that posits a normally distributed latent continuous liability to number of health pillars; latent cutoffs on the distribution determine placement of participants in the five categories [26]. A final set of models were estimated with the set of covariates listed above.

Descriptive statistics and regression tree models were performed in the statistical program R 3.5.3 [33]. All latent variable path analyses were conducted using the computer program Mplus v. 8.1 [34]. The alpha level for testing hypotheses was set to 0.05. Twin-based regression models are generally saturated, so the only source of reduced fit involves incidental issues such as differences between twins arbitrarily assigned as Twin 1 and Twin 2 within pairs. All reported models fit the data closely using standard “goodness of fit” tests.

## Results

### Descriptive statistics and intraclass correlations

Descriptive statistics for select demographic characteristics, BMI, depressive symptoms score, and the distribution of participants achieving the various health pillars are shown in Table 1. The sample was roughly 66% female and 93% White, about 60% had an annual household income over 50 K, and 79% had at least some college level education. Overall, few participants met all five health behavior standards (4.7%), with slightly more women (5.2%) achieving the five standards than men (3.8%). The 8 + h sleep criterion was met by 38.7% of the sample, whereas the 7 + h standard was met by 73.9% of the sample. Analytic results were consistent regardless of which sleep standard was used; for simplicity, we present results using the 8 + h sleep standard. Results of the *univariate* and *bivariate twin models* examining associations between each health pillar and outcome are presented in the Additional Files (pgs. 3–5, Tables A1, A2,A3,A4).

**Table 1:** Descriptive statistics of select demographic characteristics and health behaviors

	Total	Men	Women
	(*n* = 6,048 pairs)	(*n* = 2,060 pairs)	(*n* = 3,988 pairs)
Age	41.8 (*18.0*)	42.5 (*18.9*)	41.5 (*17.5*)
BMI (kg/m^2^)	25.9 (*5.6*)	26.2 (*4.6*)	25.7 (*6.1*)
Depressive Symptoms Score	0.75 (*1.21*)	0.66 (*1.11*)	0.79 (*1.25*)
Race (% White)	92.9	93.6	92.5
Health behaviors met (%)			
Sleep (8 + h)	38.7	34.9	40.6
Fruits/vegetables (5 + servings)	50.3	43.6	53.7
Sedentary time (≤ 2 h)	48.3	44.4	50.4
MVPA (150 + min)	41.2	43.1	40.3
Smoking (No)	89.8	88.7	90.4
Number of health behaviors met (%)			
0 health behaviors	1.9	2.2	1.7
1 health behavior	13.0	15.1	12.0
2 health behaviors	29.0	31.7	27.6
3 health behaviors	31.7	31.5	31.8
4 health behaviors	19.7	15.7	21.7
5 health behaviors	4.7	3.8	5.2

Continuous variables presented as mean (*standard deviation*) and categorical variables presented as percentages

### Regression tree models

#### Health pillars and BMI

Figure 1a illustrates the regression tree model when the five health pillars were used to predict BMI. The numbers in each node represent the average BMI among individuals in the corresponding subgroup, and the percentages indicate the proportion of individuals in the corresponding node. Starting from the top of the figure, the “parent” node indicates that the average BMI among all participants was 26 kg/m^2^. Participants were partitioned into three subgroups. The first subgroup (48%) included those who met the sedentary time criterion (left branch). These “non-sedentary” participants had the lowest average BMI (25 kg/m^2^). The second subgroup (20%) were those who did not meet the sedentary time criterion (middle branch) but met the MVPA criterion. These “sedentary and exercising” participants had an average BMI of 26 kg/m^2^. The third subgroup (32%) were those who did not meet the sedentary time criterion nor the MVPA criterion (right branch). These “sedentary and non-exercising” participants had the highest average BMI (27 kg/m^2^). Variable importance showed that sedentary time and MVPA were the two most important variables (variable importance = 63 and 36, respectively) in partitioning participants with different BMI. Servings of fruits and vegetables (variable importance = 1) was used as a surrogate split when there were missing data in sedentary time and/or MVPA.

#### Health pillars and depressive symptoms score

Figure 1b illustrates the regression tree model when the five health pillars were used to predict depressive symptoms score. The “parent” node indicates that the average score among all participants was 0.75 units (PHQ-2 range: 0—6). Participants were partitioned into three subgroups. The first subgroup (45%) included those who met the smoking criterion and the sedentary time criterion (left branch). These “non-smoking and non-sedentary” participants had the lowest average score (PHQ-2 = 0.54). The second subgroup (45%) were those who met the smoking criterion but did not meet the sedentary time criterion (middle branch). These “non-smoking and sedentary” participants had an average depressive symptom score of 0.82. The third subgroup (10%) were those who did not meet the smoking criterion; these “smoking” participants had the highest average depressive symptom score (PHQ-2 = 1.4). Variable importance showed that no smoking and sedentary time were the two most important variables (variable importance = 68 and 29, respectively) in partitioning participants with different depressive symptom scores. MVPA and servings of fruits and vegetables (variable importance = 2 and 1, respectively) scored very low in the variable importance measure; they were used as surrogate splits when there were missing data in the two most important variables.

### Bivariate twin analysis

#### Number of health pillars and BMI

Table 2 presents the results of the bivariate twin analyses between BMI and number of health pillars. The first set of analyses investigated the association between the number of pillars (all five health pillars) and BMI, and the second set of analyses examined the association between the number of “important” pillars (MVPA and sedentary time, from regression tree model above) and BMI. The phenotypic models (Model 1) showed significant negative relationships between health pillars and BMI (*b*_*p*_ = -0.026, *SE* = 0.003 for men; *b*_*p*_ = -0.052, *SE* = 0.003 for women, both *p*s < 0.001). Meeting an additional health pillar was associated with a 2.6% (*b*_*p*_ = -0.026, e^−.026^ = 0.97) and 5.1% (*b*_*p*_ = -0.052, e^−.052^ = 0.95) decrease in BMI for men and women, respectively.

**Table 2:** Unstandardized parameter estimates of body mass index (BMI; kg/m^2^) from the number of health pillars among same sex twins

Independent variables	All five health pillars		Two pillars (MVPA & Sedentary time)	
	Men	Women	Men	Women
Phenotypic model				
*b*_*P*_	**-.026 (.003)**	**-.052 (.003)**	**-.028 (*.004*)**	**-.061 (*.003*)**
Quasi-causal model^a^				
*b*_*P*_	-.004 (*.003*)	**-.008 (*.003*)**	**-.008 (*.004*)**	**-.018 (*.004*)**
*b*_*A*_	**-.054 (*.011*)**	**-.107 (*.011*)**	**-.050 (*.012*)**	**-.093 (*.010*)**
*b*_*C*_	**-.054 (*.011*)**	**-.107 (*.011*)**	**-.050 (*.012*)**	**-.093 (*.010*)**
Quasi-causal model^a,b^				
*b*_*P*_	**-.006 (*.002*)**	**-.006 (*.002*)**	**-.013 (*.003*)**	**-.013 (*.003*)**
*b*_*A*_	**-.051 (*.010*)**	**-.111 (*.010*)**	**-.041 (*.011*)**	**-.102 (*.009*)**
*b*_*C*_	**-.051 (*.010*)**	**-.111 (*.010*)**	**-.041 (*.011*)**	**-.102 (*.009*)**

Bolded parameter estimates are statistically significant at *p* < .05. BMI is log-transformed

*b*_*A*_ = amount of variance in body mass index attributable to additive genetic influences. *b*_*P*_ = phenotypic association between predictor and outcome. *b*_*C*_ = amount of variance in body mass index attributable to shared environmental influences

^a^ *b*_*A*_ and *b*_*C*_ are constrained to equality

^b^ *b*_*P*_ is constrained to be equal for men and women

When both additive genetic (*b*_*A*_) and shared environmental (*b*_*C*_) confounds were initially included in the model, the coefficients were estimated with large standard errors, suggesting unstable estimates and/or a lack of power to distinguish between additive genetic and shared environmental confounds. We subsequently fixed *b*_*A*_ and *b*_*C*_ to equality, meaning that between-family confounds were estimated without differentiating between genetic and shared environmental confounds. In the quasi-causal models (Model 2), the phenotypic association between health pillars and BMI remained statistically significant for women (*b*_*p*_ = -0.008, *SE* = 0.003, *p* = 0.019), but no longer significant for men (*b*_*P*_ = -0.004, *SE* = 0.003, *p* = 0.224). In Model 3, we constrained the phenotypic association between health pillars and BMI to be equal between men and women. The Wald test statistic was not statistically significant (${\chi }^{2}\left(1\right)= .580,$ *p* = 0.446), suggesting that *b*_*P*_ can be set to be the same for men and women in this model. With increased power, the quasi-causal pathway for the association between health pillars and BMI was statistically significant (*b*_*p*_ = -0.006, *SE* = 0.002, *p* = 0.011). This association reflected a < 1% (e^−.006^ = 0.99) decrease in BMI for each additional health pillar met, suggesting minimal relation between health pillars and BMI after accounting for between-family confounds. This quasi-causal association between health pillars and BMI was no longer significant (*b*_*p*_ = -0.004, *SE* = 0.002, *p* = 0.086) after controlling for participants’ sociodemographic characteristics (Table 3).

**Table 3:** Unstandardized parameter estimates of body mass index (BMI; kg/m^2^) from the number of health pillars among same sex twins (with covariates)

Independent variables	All five health pillars		Two pillars (MVPA & Sedentary time)	
	Men	Women	Men	Women
Quasi-causal model^a,b^				
*b*_*P*_	-.004 (*.002*)	-.004 (*.002*)	**-.011 (*.002*)**	**-.011 (*.002*)**
*b*_*A*_	**-.039 (*.010*)**	**-.089 (*.010*)**	**-.027 (*.011*)**	**-.081 (*.010*)**
*b*_*C*_	**-.039 (*.010*)**	**-.089 (*.010*)**	**-.027 (*.011*)**	**-.081 (*.010*)**
Covariates				
Age	**.031 (*.002*)**	**.029 (*.002*)**	**.030 (*.002*)**	**.028 (*.002*)**
Race	.020 (*.014*)	**-.022 (*.010*)**	.022 (*.014*)	**-.020 (*.010*)**
Income	.001 (*.001*)	**-.012 (*.001*)**	.002 (*.001*)	**-.011 (*.001*)**
Education	**-.011 (*.004*)**	**-.026 (*.004*)**	**-.009 (*.004*)**	**-.024 (*.004*)**

Bolded parameter estimates are statistically significant at *p* < .05. BMI is log-transformed

*b*_*P*_ = phenotypic association between predictor and outcome. *b*_*A*_ = amount of variance in body mass index attributable to additive genetic influences. *b*_*C*_ = amount of variance in body mass index attributable to shared environmental influences

^a^ *b*_*A*_ and *b*_*C*_ are constrained to equality

^b^ *b*_*P*_ is constrained to be equal for men and women

The phenotypic effect of health pillars on BMI is illustrated in Fig. 2 (top panel). We computed the average difference in BMI across individuals who met different numbers of health pillars (e.g., a one-unit difference in health pillars met reflect the comparison between those who met one versus zero, two versus one, three versus two, four versus three, and five versus four). The average difference in BMI across all comparisons was negative, with the average difference in BMI increases with increased differences in health pillars met, reflecting that participants who endorsed more health pillars were more likely to have lower average BMIs than those who endorsed fewer health pillars. The bottom panel of Fig. 2 illustrates the average within-pair difference in BMI among twin pairs who differ in the number of health pillars met. Note that no twin pairs had five-units difference in health pillars (i.e., one twin met five pillars, co-twin did not meet any pillars), therefore no bars were present at five units. Consistent across twin pairs with varying within-pair differences in health pillars, there is a very small within-pair difference in BMI. This means that within a pair of twins who differ in the number of health pillars met, the member of the twin pair who met more health pillars has slightly lower BMI than their co-twin who met fewer health pillars. However, the average within-pair difference in BMI was very small; a difference in three health pillars met is associated with < 1 kg/m^2^ difference in BMI (third set of bars from the left). Although the average within-pair difference in BMI appears to be larger among twin pairs with four units of difference in health pillars met (i.e., one twin met all five health pillars, co-twin met one health pillars; or one twin met four pillars, co-twin did not meet any pillars; fourth set of bars from the left), the number of twin pairs in this group was small (*n* = 16 and 14 for MZ and DZ pairs, respectively), with large standard errors in the average within-pair difference in BMI.

Results were mostly similar when only MVPA and sedentary time, the two health pillars found to be the most important pillars from the regression tree analyses, were used. In Model 1, there were significant negative associations between health pillars and BMI (*b*_*p*_ = -0.028, *SE* = 0.004 for men; *b*_*p*_ = -0.061, *SE* = 0.003 for women, both *p*s < 0.001) (Table 2). When between-family confounds were included (Model 2), the phenotypic association between health pillars and BMI remained statistically significant (*b*_*p*_ = -0.008, *SE* = 0.004, *p* = 0.017 for men; *b*_*p*_ = -0.018, *SE* = 0.004, *p* < 0.001 for women). Results remained consistent when the phenotypic association between health pillars and BMI was constrained to be equal between men and women in Model 3 (*b*_*p*_ = -0.013, *SE* = 0.003, *p* < 0.001), and reduced but remained significant after controlling for covariates (*b*_*p*_ = -0.011, *SE* = 0.002, *p* < 0.001) (Table 3).

**Fig. 3:**
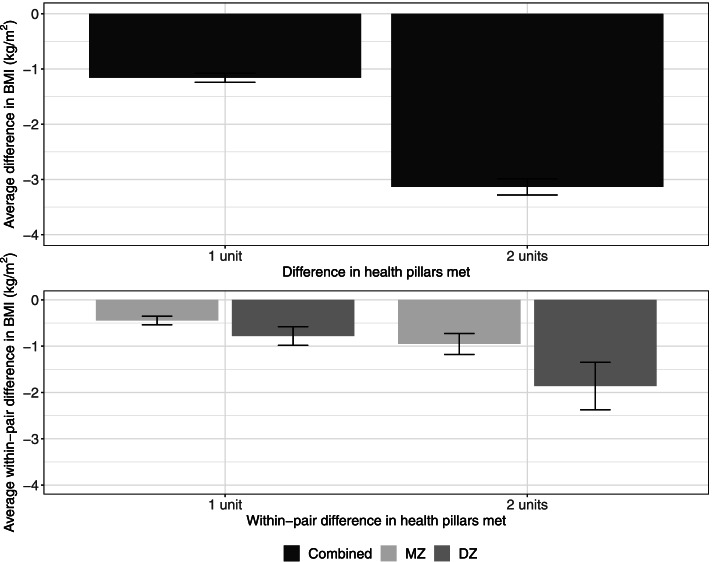
Average difference in BMI across individuals meeting different number of health pillars (MVPA and sedentary time only) (top panel) and average within-pair difference in BMI between twin pairs meeting different number of health pillars by zygosity (MVPA and sedentary time only) (bottom panel). Error bars denote standard errors

As shown in the top panel of Fig. 3, the average difference in BMI increases with an increased difference in the number of health pillars met, reflecting that the average BMI is lower among individuals meeting more pillars than those meeting fewer pillars. The average within-pair difference in BMI among twin pairs who differed in the number of health pillars met is illustrated in the bottom panel of Fig. 3. We observed a slightly larger average within-pair difference in BMI among twin pairs with two units of difference in health pillars met (i.e., one twin met both pillars, and co-twin met none of the two pillars; right bars) than among those with one unit of difference in health pillars met (i.e., one twin met both pillars and co-twin met one pillar, or one twin met one pillar and co-twin met none; left bars).

#### Number of health pillars and depressive symptoms score

Results of the bivariate twin analyses between depressive symptoms score and number of health pillars are shown in Table 4. The phenotypic model (Model 1) showed negative relationships between health pillars (all five pillars) and depressive symptom score (*b*_*p*_ = -0.120, *SE* = 0.013 for men; *b*_*p*_ = -0.168, *SE* = 0.010 for women, both *p*s < 0.001), though the effect was very small.

**Table 4:** Unstandardized parameter estimates of depressive symptom score from the number of health pillars among same sex twins

Independent variables	All five health pillars		Two pillars (Sedentary time & Smoking)	
	Men	Women	Men	Women
Phenotypic model				
*b*_*P*_	**-.120 (*.013*)**	**-.168 (*.010*)**	**-.146 (*.014*)**	**-.166 (*.011*)**
Quasi-causal model^a^				
*b*_*P*_	**-.075 (*.019*)**	**-.112 (*.015*)**	**-.079 (*.025*)**	**-.127 (*.020*)**
*b*_*A*_	**-.109 (*.051*)**	**-.133 (*.037*)**	**-.116 (*.047*)**	-.070 (*.038*)
*b*_*C*_	**-.109 (*.051*)**	**-.133 (*.037*)**	**-.116 (*.047*)**	-.070 (*.038*)
Quasi-causal model^a,b^				
*b*_*P*_	**-.098 (*.012*)**	**-.098 (*.012*)**	**-.110 (*.015*)**	**-.110 (*.025*)**
*b*_*A*_	-.062 (*.039*)	**-.161 (*.033*)**	-.067 (*.035*)	**-.097 (*.032*)**
*b*_*C*_	-.062 (*.039*)	**-.161 (*.033*)**	-.067 (*.035*)	**-.097 (*.032*)**

Bolded parameter estimates are statistically significant at *p* < .05. Depressive symptom score is square root transformed

*b*_*P*_ = phenotypic association between predictor and outcome. *b*_*A*_ = amount of variance in body mass index attributable to additive genetic influences. *b*_*C*_ = amount of variance in body mass index attributable to shared environmental influences

^a^ *b*_*A*_ and *b*_*C*_ are constrained to equality

^b^ *b*_*P*_ is constrained to be equal for men and women

In Model 2, the between-family confounds were estimated rather than individual additive genetic (*b*_*A*_) and shared environmental (*b*_*C*_) confounds, as described previously. The phenotypic association between health pillars and depressive symptoms remained significant (*b*_*P*_ = -0.075, *SE* = 0.019 for men; *b*_*P*_ = -0.112, *SE* = 0.015 for women; both *p*s < 0.001), after controlling for between-family confounds. In Model 3, we constrained the phenotypic association between health pillars and depressive symptom score to be equal between men and women. The Wald test statistic was not statistically significant (${\chi }^{2}\left(1\right)= 2.307,$ *p* = 0.129), suggesting that *b*_*P*_ can be set to be the same for men and women in this model. With increased power, the quasi-causal pathway for the association between health pillars and depressive symptom score remained statistically significant (*b*_*p*_ = -0.098, *SE* = 0.012, *p* < 0.001). The quasi-causal pathway was reduced (*b*_*p*_ = -0.086, *SE* = 0.012, *p* < 0.001), but remained statistically significant after controlling for covariates (Table 5).

**Table 5:** Unstandardized parameter estimates of depressive symptoms score from the number of health pillars among same sex twins (with covariates)

Independent variables	All five health pillars		Two pillars (MVPA & Sedentary time)	
	Men	Women	Men	Women
Quasi-causal model^a,b^				
*b*_*P*_	**-.086 (*.012*)**	**-.086 (*.012*)**	**-.088 (*.015*)**	**-.088 (*.015*)**
*b*_*A*_	**-.080 (*.040*)**	**-.148 (*.035*)**	-.070 (*.037*)	**-.090 (*.035*)**
*b*_*C*_	**-.080 (*.040*)**	**-.148 (*.035*)**	-.070 (*.037*)	**-.090 (*.035*)**
Covariates				
Age	**-.026 (*.006*)**	**-.029 (*.005*)**	**-.025 (*.006*)**	**-.031 (*.005*)**
Race	.006 (*.050*)	-.033 (*.032*)	.016 (*.050*)	-.032 (*.031*)
Income	**-.036 (*.005*)**	**-.043 (*.004*)**	**-.031 (*.005*)**	**-.041 (*.004*)**
Education	-.018 (*.016*)	**-.032 (*.012*)**	-.003 (*.017*)	**-.019 (*.013*)**

Bolded parameter estimates are statistically significant at *p* < .05. Depressive symptom score is square root transformed

*b*_*P*_ = phenotypic association between predictor and outcome. *b*_*A*_ = amount of variance in body mass index attributable to additive genetic influences. *b*_*C*_ = amount of variance in body mass index attributable to shared environmental influences

^a^ *b*_*A*_ and *b*_*C*_ are constrained to equality

^b^ *b*_*P*_ is constrained to be equal for men and women

**Fig. 4:**
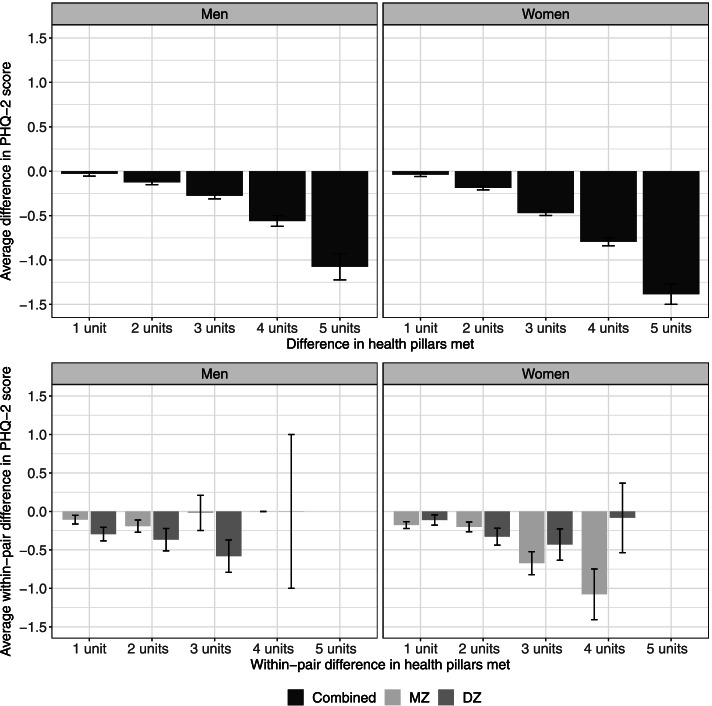
Average difference in PHQ-2 score across individuals meeting different number of health pillars (top panel) and average within-pair difference in PHQ-2 score between twin pairs meeting different number of health pillars by zygosity (bottom panel). Error bars denote standard errors

We illustrate the phenotypic effect of health pillars on depressive symptom score in Fig. 4 (top panel). The average difference in depressive symptom score increases with increased difference in health pillars met, reflecting that participants who endorsed more health pillars were more likely to have a lower average depressive symptom score than those who endorsed fewer health pillars, with the effect slightly larger among women (right panel) than men (left panel). The bottom panel of Fig. 4 illustrates the average within-pair difference in depressive symptom score among twin pairs who differ in the number of health pillars met. As previously described, no twin pairs had five-units difference in health pillars, therefore no bars were present at five units. We observed a very slight increase in the within-pair difference in depressive symptom score with increasing within-pair difference in health pillars; co-twins who met more health pillars were more likely to have lower depressive symptom score than their co-twin who met fewer health pillars. However, even within twin pairs with three-units difference in health pillars, there is only less than one-unit difference in depressive symptom score (third set of bars from the left).

Results were similar when only sedentary time and smoking, the two health pillars found to be the most important from the regression tree analyses, were used. In Model 1, there were significant negative associations between health pillars and depressive symptom score (*b*_*p*_ = -0.146, *SE* = 0.014 for men; *b*_*p*_ = -0.166, *SE* = 0.011 for women, both *p*s < 0.001) (Table 4). When between-family confounds were included (Model 2), the phenotypic association between health pillars and depressive symptom score remained statistically significant (*b*_*p*_ = -0.079, *SE* = 0.025, *p* = 0.001 for men; *b*_*p*_ = -0.127, *SE* = 0.020, *p* < 0.001 for women), and when phenotypic association between health pillars and depressive symptom score was constrained to be equal between men and women in Model 3 (*b*_*p*_ = -0.110, *SE* = 0.015, *p* < 0.001). Results were consistent when further controlling for covariates (*b*_*p*_ = -0.088, *SE* = 0.015, *p* < 0.001) (Table 5).

**Fig. 5:**
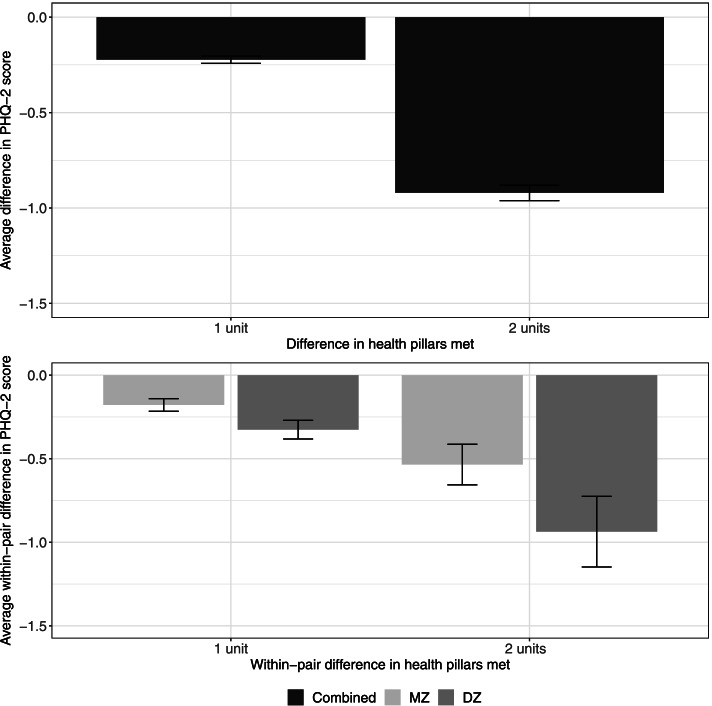
Average difference in PHQ-2 score across individuals meeting different number of health pillars (sedentary time and non-smoking only) (top panel) and average within-pair difference in PHQ-2 score between twin pairs meeting different number of health pillars by zygosity (sedentary time and non-smoking only) (bottom panel). Error bars denote standard errors

As shown in Fig. 5 (top panel), the average difference in depressive symptoms score increases with an increased difference in number of health pillars met, reflecting that individuals meeting more pillars have, on average, a lower depressive symptoms score than those meeting fewer pillars. The average within-pair difference in depressive symptoms among twin pairs who differ in the number of health pillars met is illustrated in Fig. 5 (bottom panel). We observed a slightly larger average within-pair difference in depressive symptoms among twin pairs with two units’ difference in health pillars met (i.e., one twin met both pillars, and co-twin met none of the two pillars; set of bars on the right) than among those with one unit difference (i.e., one twin met both pillars and co-twin met one, or one twin met one pillar and co-twin met none; set of bars on the left).

## Discussion

This study examined the association between achievement of key health behaviors with BMI and depressive symptoms. In general, the results of this study support our hypotheses that achievement of more health behaviors would be associated with beneficial health outcomes, indicated by lower BMI and lower depressive symptoms score. Overall, few participants met all five health behavior pillars (less than 5%), with slightly more women achieving the five pillars than men. These findings are consistent with other reports investigating achievement of health behaviors using nationally representative data [9, 11, 12], although the behaviors examined differ across studies. While each of the five pillars of health should continue to be promoted for general health and well-being, for those struggling to meet multiple health patterns, which represents a substantial portion of the population, initial targeting of key behaviors with potential for greater impact on a desired outcome may improve efficacy and long-term lifestyle adaptation. Specifically, targeting sedentary time and MVPA may have the greatest impact on BMI, whereas targeting smoking and sedentary time may have the greatest impact on depressive symptoms.

### Health pillars and BMI

The present findings indicated a significant association between achievement of health behaviors and BMI. In general, there was a dose–response pattern such that participants who endorsed more health behaviors had lower average BMI values than those who endorsed fewer health behaviors (see Fig. 2 top panel). Among the five health pillars investigated, we found sedentary and MVPA standards were the most influential for BMI. When only sedentary time and MVPA were considered, we showed there exists a “quasi-causal” association between the number of health pillars (zero to meeting both sedentary time and MVPA pillars) and BMI, after taking into account between-family factors and demographic covariates. Our findings provide cross-sectional evidence suggesting that even endorsement of these two important health pillars may have a positive impact on BMI. Importantly, these results are consistent with previous prospective cohort studies examining the impact of combinations of healthy lifestyle behaviors on mortality [35]. In another longitudinal study, Li and colleagues reporting that adherence to five low-risk lifestyle-related factors (not smoking, a healthy weight, regular physical activity, a healthy diet, and moderate alcohol consumption) could prolong life expectancy, compared with individuals who adopted no low-risk lifestyle factors [36]. Others studies using isotemporal substitution modeling have also supported the importance of physical activity-related behaviors for obesity and cardiovascular health. German and colleagues reported that replacing sedentary time with MVPA was associated with more desirable cardiometabolic risk profiles [37]. Additionally, Buman et al. [38], reported a powerful influence of MVPA and sedentary time on disease risk biomarkers (including waist circumference as a measure of obesity).

### Health pillars and depressive symptoms score

The pattern of associations between health behaviors and depressive symptoms were remarkably consistent with that between health behaviors and BMI (see Tables 4 and 5). Sedentary and smoking standards were the most influential for depressive symptoms; the average depression symptoms score was lower among individuals who met both these health pillars, compared to those who met one or none. This association, though small, remained robust after taking into account between-family influences and confounding demographic factors. While sedentary time is associated with a myriad of chronic and cardiometabolic disease states, it is of particular importance for independently predicting depression; a previous longitudinal study supports this being due to loss of social engagement and higher screen time (television viewing and computer use) typically incurred through a sedentary lifestyle [39]. On the other hand, smoking is hypothesized to exacerbate depressive symptoms in those already experiencing poor mental health and seeking short-term alleviating effects of tobacco over time, a phenomena known as the “self-medication model” [40, 41].

The present study posits that, independent of genetic and shared environmental factors, achieving specific health recommendations in combination (i.e., sedentary time, MVPA, and smoking) may have varying benefits depending on the targeted health outcome (BMI versus depressive symptoms). Given these findings in combination with previous longitudinal studies described above, and considering overall difficulty in achieving health recommendations for the US population, future research informing strategies to improve health should utilize multifactorial approaches. Further, findings that any combination of achieving various recommended health behaviors yield some health benefit should guide practitioner recommendations when prescribing behavior changes in a clinical and/or public health setting.

### Strengths and limitations

This study examined associations between highly promoted health behaviors with health outcomes in a genetically informed community-based statewide sample of U.S. adults. An important strength is use of the twin design to re-examine general “population-level” associations that have been reported in the literature; these phenotypic associations remained after taking into account between-family confounds shared within twin pairs, thus increasing the confidence in interpreting observed associations as “quasi-causal” rather than purely observational. We had a relatively large sample of identical and fraternal twins with complete data, leading to robust estimates free of bias from imputation methods.

On the other hand, the study relied on self-report measures, which are known to result in measurement bias. However, we do provide internal validation metrics for BMI and MVPA, thus increasing the confidence in these measures. Average depressive symptom score of this sample is relatively low, and thus may have been biased in that those experiencing lower anxiety/stress were more likely to participate in the survey. For these reasons, and due to the sample being primarily White (92.9%), generalizability of the study findings is limited. Finally, although the twin design can control for between-family confounds, the cross-sectional design precludes definitive causal inferences, especially as regards the possibility of reverse causation. A longitudinal design investigating changes in health behaviors and changes in BMI and depressive symptoms scores, between and within twins, would allow for more definitive conclusions to be drawn about direction of effect. Nonetheless, our findings are consistent with findings from prospective cohort studies demonstrating that health behaviors “drive” health outcomes, not the other way around as might be suggested by reverse causation.

## Conclusions

The present study demonstrates that achievement of commonly endorsed health behaviors, independently and in specific combinations, is associated with lower BMI and depressive symptoms both between and within-twin pairs. These findings suggest a need for future longitudinal data to ultimately inform behavioral interventions to optimizing health behaviors most important to improving the specific targeted health outcomes. A negative dose–response relationship between achieving these pillars of health was observed in relation to BMI and depressive symptoms. Regression tree analysis demonstrated that sedentary time and MVPA were the behavioral targets most correlated with reduced BMI, whereas sedentary time and smoking yielded the strongest associations with lower depressive symptoms. Thus, while achieving each of the five pillars of health are related to higher levels of general health and well-being, for those struggling to meet multiple health patterns, initial targeting of those behaviors with highest correlation with a desired outcome (i.e., weight versus mental health) may improve efficacy of behavior change efforts. Future longitudinal studies and interventions should seek to understand whether pursuit of specific combinations of health behaviors yield greater feasibility and/or effectiveness to improve health outcomes over time.

## Supplementary Information

**Additional file 1. **Appendix Material.

## Data Availability

The data supporting the results of the present study are owned by the Washington State Twin Registry (WSTR). Thus, the data cannot be publicly shared as it involves third party data. However, researchers interested to apply to gain access to the data can do so by contacting the WSTR and completing the appropriate forms stipulated in the WSTR Policies & Procedures guidelines. Application information can be sent to the Scientific Operations Manager at the following URL (https://wstwinregistry.org/contact-us/) or via email (ws.twinregistry@wsu.edu).

## Abbreviations

BMI: Body mass index
CART: Classification and regression tree
DZ: Dizygotic
FIML: Full information maximum likelihood
MVPA: Moderate-to-vigorous physical activity
MZ: Monozygotic
PHQ-2, 2: Item Patient Health Questionnaire-2
WSTR: Washington State Twin Registry
