# Supplementary material for: The pillars of health: influence of multiple lifestyle behaviors on body mass index and depressive symptoms in adult twins
Source: BMC Public Health. 2022 Aug 5;22:1487. doi: 10.1186/s12889-022-13901-7 (PMC9354427; doi:10.1186/s12889-022-13901-7)
Supplement: Supplementary file 1 — Additional file 1. Appendix Material. [file 12889_2022_13901_MOESM1_ESM.zip › Pillarsofhealth_AdditionalFile R1.pdf]

Appendix Material

Contents **Page**

Appendix – Methods

A1. Univariate twin model description ………………………………………………………………. 2

A2. Bivariate twin model description ……………………………………………………………... 2

Appendix – Results

A3. Univariate twin model analyses …………………………………………………………...…3

A4. Bivariate twin model analyses …………………………………………………………………4

Appendix – Tables

Table A1. Twin intraclass correlations and standardized variance components for body mass index (BMI), depressive symptom score, and the number of health pillars achieved ……………….…………6

Table A2. Twin intraclass correlations and standardized variance components for each health pillar achieved ...……………………………...…………………………………………………7

Table A3. Unstandardized parameter estimates of body mass index (BMI; kg/m2) from each health pillar among same sex twins ….………………………………………………………………………8

Table A4. Unstandardized parameter estimates of self-reported depressive symptom score from each health pillar among same sex twins ……………………………………………………….……9

Appendix – Figure Legends

Figure A1. Classical univariate twin model for one variable ……………………………………………10

Figure A2. Path diagram of a bivariate twin model (only one twin shown for clarity) …….…………....10

Appendix – Methods

A1. Univariate twin model description

We first used the classical twin model to decompose the variances of BMI, depressive symptoms, each of the five health pillars (sleep 8h+, 5+ servings of fruits and vegetables, <2h sedentary time, ≥150 min MVPA, and no smoking), and the number of health pillars into three components: additive genetic (A), shared environmental (C), and non-shared environmental (E) factors (Figure A1). The A variance components represent the additive effect of genes; it correlates $r = 1.0$ between MZ twins (who share 100% of their genetic sequence) and $r = 0.5$ between DZ twins (who share 50% of their segregating genes, on average). The C variance components represent common environmental experiences that make members of the family more similar; they correlate at $r = 1.0$ for both MZ and DZ twins. The E variance components represent unique environmental experiences and do not correlate between twins. The E variance components also include measurement error.

A2. Bivariate twin model description

Next, we utilized the bivariate twin model to examine the relation between the endorsement of each health pillar (sleep 8h+, 5+ servings of fruits and vegetables, <2h sedentary time, ≥150 min MVPA, and no smoking) and health outcomes (BMI or depressive symptoms). Because the achievement of each health pillar is not randomly assigned, one cannot be sure that the phenotypic association between the health pillar achieved, and the health outcomes of interest is the result of a causal effect. This phenotypic relationship may occur due to genetic predispositions that are associated with the number of health behaviors achieved also leading to better health outcome (i.e., lower BMI and depressive symptoms), or due to shared environmental factors (e.g., higher socioeconomic status during childhood) that predispose to the achievement of health pillar and better health outcomes.

Twin designs are especially useful for understanding measured and unmeasured uncontrolled confounds in non-experimental data. If the effect of health behaviors on health outcomes is truly causal, then one would expect it to manifest both between twin pairs (pairs where both endorsed a particular health pillar would have lower average BMI or depressive symptom score than pairs where neither endorsed the health pillar) and within pairs (the member of a pair who endorsed a particular health pillar would have lower BMI/depressive symptoms

than the co-twin who did not endorse the same health pillar). If, however, the association is the result of uncontrolled confounding variables such as genetic background or socioeconomic status, the association will be observed between pairs but not within them, because twin pairs share a rearing environment and either all or half of their genetic background. The twin method cannot fully control for all potential confounds, however, and some uncontrolled variables may vary within pairs as well as between them. We therefore refer to associations that have survived genetically informed tests as "quasi-causal," to suggest that the twin analysis has strengthened our confidence in the causal underpinning of the association. Details of the bivariate twin model and the statistical methods are described in Turkheimer & Harden (Turkheimer E, Harden KP. Behavior genetic research methods: Testing quasi-causal hypotheses using multivariate twin data. In: Reis HT, Judd CM, eds. Handbook of Research Methods in Social and Personality Psychology. 2nd ed. Cambridge, UK: Cambridge University Press; 2014:

In Figure A2, we use the association between the endorsement of a health pillar (sleep 8h+) and BMI as an example to illustrate the bivariate twin model. The health pillar and BMI are both partitioned into ACE components using the classical twin method. In addition, BMI is regressed on phenotypic health behaviors (b_P), as well as on the shared components (b_A and b_C) of the health pillar. In Model 1, b_A and b_C are set to zero, leaving a simple regression of BMI on health behaviors at the individual level. This is a phenotypic association model; it tests for the association of the health pillar with BMI without including genetic or shared environmental confounds. The model is then re-estimated including estimates of b_A and b_C, which controls for genetic and shared environmental confounds, respectively, in the estimation of the phenotypic effect. This is referred to as a quasi-causal model (Model 2). In Model 3, the estimated parameters for men and women are constrained to be equal. Similar sets of analyses were performed to examine the association between the remaining four health pillars and BMI, as well as between each health pillar and depressive symptoms score.

Appendix – Results

A3. Univariate twin model analyses

Table A1 shows the twin correlations and standardized biometric variance for BMI, depressive symptoms, and number of health pillars. For BMI, most of the variance was attributable to the additive genetic component (71% and 69% for men and women, respectively),

with a smaller proportion attributable to non-shared environment (26% and 23% for men and women, respectively), and a very small proportion attributable to shared environment (3% and 9% for men and women, respectively). For depressive symptoms, most of the variance was attributable to unique environment (72% and 67% for men and women, respectively), with a smaller proportion attributable to the additive genetic component (28% and 26% for men and women, respectively), and a very small proportion attributable to shared environment (0% and 7% for men and women, respectively). For the number of health pillars, most of the variance was attributable to unique environment (65% and 64% for both men and women, respectively), with a smaller proportion attributable to the additive genetic component (23% and 31% for men and women, respectively) and shared environment (12% and 6% for men and women, respectively).

Table A2 shows the tetrachoric twin correlations and standardized biometric variance for each health pillar. All shared environmental components (C) are fixed to zero. Except for smoking, larger proportions of the variance of each of the remaining four pillars was attributable to non-shared environment, with smaller proportions attributable to additive genetic component.

A4. Bivariate twin model analyses

Health pillars and BMI. Table A3 presents the results of the bivariate twin analyses between BMI and each health pillar. The phenotypic models (Models 1) showed significant negative associations between BMI and each health pillar, except for servings of fruits and vegetables and no smoking for men. Meeting the MVPA criteria is associated with the largest decrease in BMI (b_p = -.031; $e^{-.031}$ = .97, approximately 3% decrease) for men. Meeting the sedentary time criteria is associated with the largest decrease in BMI (b_p = -.056; $e^{-.056}$ = .95, approximately 5.4% decrease) for women.

When additive genetic confounds are controlled in the quasi-causal models (Models 2), the phenotypic association between the health pillar and BMI remained statistically significant only for sedentary time (b_p = -.009, SE = .004, p = .035), MVPA (b_p = -.018, SE = .004, p < .001), and smoking (b_p = .065, SE = .014, p < .001) for women. In Model 3, we constrained the phenotypic association between each health pillar and BMI to be equal between men and women. The Wald test statistic was not statistically significant for the sleep ($\chi^2(1) = .007$, p = .934), fruits and vegetables ($\chi^2(1) = .223$, p = .637), sedentary time ($\chi^2(1) = .112$, p = .738), and smoking ($\chi^2(1) = 1.119$, p = .290) pillar, suggesting that b_P can be set to be the same for

men and women in these four models. With increased power, the quasi-causal pathway for the association between health pillar and BMI was statistically significant for fruits and vegetables (b_p = -.007, SE = .003, p = .010), sedentary time (b_p = -.008, SE = .003, p = .012), and smoking (b_p = -.056, SE = .010, p < .001).

Health pillars and depressive symptoms. Results of the bivariate twin analyses between depressive and each health pillar are shown in Table A4. The phenotypic models (Models 1) showed significant negative associations between depressive symptoms and each health pillar, except for sleep and servings of fruits and vegetables for men. Meeting the no smoking criteria is associated with the largest decrease in depressive symptoms for both men (b_p = -.148, SE = .018, p < .001) and women (b_p = -.181, SE = .014, p < .001), though the difference was very small (less than .5 unit decrease in depressive symptoms out of a total possible score of 6, compared to those who did not meet the no smoking criteria).

When additive genetic confounds are controlled in the quasi-causal models (Models 2), the phenotypic association between the health pillar and depressive symptoms remained statistically significant except for sleep, MVPA, and no smoking for men. In Model 3, we constrained the phenotypic association between each health pillar and depressive symptoms to be equal between men and women. The Wald test statistic was not statistically significant for the sleep ($\chi^2(1) = 3.068$, p = .080), fruits and vegetables ($\chi^2(1) = .001$, p = .970), sedentary time ($\chi^2(1) = .231$, p = .631), and MVPA ($\chi^2(1) = .868$, p = .352), suggesting that b_P can be set to be the same for men and women in these four models. With increased power, the quasi-causal pathway for the association between health pillar and depressive symptoms was statistically significant for all four models.

Table A1. Twin intraclass correlations and standardized variance components for body mass index (BMI), depressive symptoms score, and the number of health pillars achieved.

	Men		Women	
	Estimate	(SE)	Estimate	(SE)
BMI (kg/m^2)				
*r*MZ	**.74**	**(.01)**	**.77**	**(.01)**
rDZ	**.39**	**(.03)**	**.43**	**(.02)**
a^2	**.71**	**(.07)**	**.69**	**(.04)**
c^2	.03	(.06)	.09	(.04)
e^2	**.26**	**(.01)**	**.23**	**(.01)**
Depressive symptoms				
*r*MZ	**.28**	**(.02)**	**.33**	**(.02)**
rDZ	**.14**	**(.01)**	**.20**	**(.03)**
a^2	**.28**	**(.02)**	**.26**	**(.06)**
c^2	0	(.004)	.07	(.06)
e^2	**.72**	**(.02)**	**.67**	**(.02)**
Number of health pillars				
*r*MZ	**.34**	**(.03)**	**.35**	**(.02)**
rDZ	**.22**	**(.04)**	**.20**	**(.03)**
a^2	.23	(.10)	**.31**	**(.07)**
c^2	.12	(.10)	.06	(.06)
e^2	**.65**	**(.03)**	**.64**	**(.02)**

SE = standard error. *r*MZ = correlations between monozygotic (MZ) twins. *r*DZ = correlations between dizygotic (DZ) twins. a^2 = additive genetic variance. c^2 = shared-environmental variance. e^2 = non-shared environmental variance. Bolded parameter estimates are statistically significant at $p < .05$.

Table A2. Twin intraclass correlations and standardized variance components for each health pillar achieved.

	Men		Women	
	Estimate	(SE)	Estimate	(SE)
Sleep				
*r*MZ	**.43**	**(*.04*)**	**.39**	**(*.03*)**
rDZ	**.22**	**(*.02*)**	**.20**	**(*.01*)**
a^2	**.43**	**(*.04*)**	**.39**	**(*.03*)**
c^2	-	-	-	-
e^2	**.57**	**(*.04*)**	**.61**	**(*.03*)**
Fruits & vegetables				
*r*MZ	**.28**	**(*.04*)**	**.32**	**(*.03*)**
rDZ	**.14**	**(*.02*)**	**.16**	**(*.01*)**
a^2	**.28**	**(*.04*)**	**.32**	**(*.03*)**
c^2	-	-	-	-
e^2	**.73**	**(*.04*)**	**.68**	**(*.03*)**
Sedentary time				
*r*MZ	**.41**	**(*.04*)**	**.46**	**(*.03*)**
rDZ	**.21**	**(*.02*)**	**.23**	**(*.01*)**
a^2	**.41**	**(*.04*)**	**.46**	**(*.03*)**
c^2	-	-	-	-
e^2	**.59**	**(*.04*)**	**.54**	**(*.03*)**
MPVA				
*r*MZ	**.35**	**(*.04*)**	**.36**	**(*.03*)**
rDZ	**.17**	**(*.02*)**	**.18**	**(*.01*)**
a^2	**.35**	**(*.04*)**	**.36**	**(*.03*)**
c^2	-	-	-	-
e^2	**.65**	**(*.04*)**	**.64**	**(*.03*)**
Smoking				
*r*MZ	**.78**	**(*.03*)**	**.74**	**(*.03*)**
rDZ	**.39**	**(*.02*)**	**.37**	**(*.01*)**
a^2	**.78**	**(*.03*)**	**.74**	**(*.03*)**
c^2	-	-	-	-
e^2	**.22**	**(*.03*)**	**.26**	**(*.03*)**

SE = standard error. Sleep = met criteria of sleep 8+ h or not. Fruits & vegetables = met criteria of 5+ servings or not. Sedentary time = met criteria of < 2h sedentary time or not. MVPA = met criteria of 150min MVPA per week or not. Smoking = met criteria of not smoking or not. *r*MZ = correlations between monozygotic (MZ) twins. *r*DZ = correlations between dizygotic (DZ) twins. a^2 = additive genetic variance. c^2 = shared-environmental variance; fixed to zero. e^2 = non-shared environmental variance. Bolded parameter estimates are statistically significant at $p < .05$.

Table A3. Unstandardized parameter estimates of body mass index (BMI; kg/m2) from each health pillar among same sex twins.

Independent variables	Sleep		Fruits & vegetables		Sedentary time		MVPA		Smoking	
	Men	Women	Men	Women	Men	Women	Men	Women	Men	Women
Phenotypic model										
b_P	**-.021 (*.004*)**	**-.016 (*.004*)**	-.006 (*.004*)	**-.015 (*.004*)**	**-.015 (*.004*)**	**-.056 (*.004*)**	**-.031 (*.004*)**	**-.045 (*.004*)**	0 (*.005*)	**-.029 (*.005*)**
Quasi-causal model										
b_P	.004 (*.005*)	.004 (*.004*)	**-.008 (*.004*)**	-.006 (*.004*)	-.007 (*.005*)	**-.009 (*.004*)**	-.006 (*.004*)	**-.018 (*.004*)**	**.043 (*.015*)**	**.065 (*.014*)**
b_A	**-.051 (*.013*)**	**-.043 (*.013*)**	.009 (*.017*)	-.024 (*.014*)	-.016 (*.013*)	**-.089 (*.012*)**	**-.062 (*.015*)**	**-.067 (*.014*)**	**-.051 (*.019*)**	**-.115 (*.018*)**
Quasi-causal model[a]										
b_P	.004 (*.003*)	.004 (*.003*)	**-.007 (*.003*)**	**-.007 (*.003*)**	**-.008 (*.003*)**	**-.008 (*.003*)**	-	-	**.056 (*.010*)**	**-.056 (*.010*)**
b_A	**-.051 (*.011*)**	**-.044 (*.011*)**	.005 (*.015*)	-.021 (*.012*)	-.014 (*.011*)	**-.091 (*.010*)**	-	-	**-.065 (*.013*)**	**-.105 (*.014*)**

Bolded parameter estimates are statistically significant at $p < .05$. BMI is log-transformed.

Sleep = met criteria of sleep 8+h or not. Fruits & vegetables = met criteria of 5+ servings or not. Sedentary time = met criteria of <2h sedentary time or not. MVPA = met criteria of 150min MVPA per week or not. Smoking = met criteria of not smoking or not.

b_P = phenotypic association between predictor and outcome. b_A = amount of variance in body mass index attributable to additive genetic influences.

[a] b_P is constrained to be equal for men and women; this model was not performed for MVPA as Wald test showed b_p to be statistically significantly different between men and women (Wald = 4.279, p = .039).

Table A4. Unstandardized parameter estimates of self-reported depressive symptoms score from each health pillar among same sex twins.

Independent variables	Sleep		Fruits & vegetables		Sedentary time		MVPA		Smoking	
	Men	Women	Men	Women	Men	Women	Men	Women	Men	Women
Phenotypic model										
b_P	-.022 (*.014*)	**-.040 (*.011*)**	-.024 (*.014*)	**-.086 (*.011*)**	**-.117 (*.015*)**	**-.126 (*.012*)**	**-.064 (*.015*)**	**-.119 (*.012*)**	**-.148 (*.018*)**	**-.181 (*.014*)**
Quasi-causal model										
b_P	.008 (*.028*)	**-.051 (*.019*)**	**-.060 (*.022*)**	**-.061 (*.018*)**	**-.079 (*.028*)**	**-.096 (*.022*)**	-.040 (*.024*)	**-.069 (*.019*)**	-.080 (*.071*)	**-.269 (*.056*)**
b_A	-.060 (*.059*)	.024 (*.045*)	.114 (*.073*)	-.068 (*.050*)	-.080 (*.060*)	-.058 (*.044*)	-.060 (*.063*)	**-.121 (*.049*)**	-.080 (*.087*)	.109 (*.070*)
Quasi-causal model[a]										
b_P	**-.031 (*.016*)**	**-.031 (*.016*)**	**-.061 (*.014*)**	**-.061 (*.014*)**	**-.089 (*.017*)**	**-.089 (*.017*)**	**-.058 (*.015*)**	**-.058 (*.015*)**	-	-
b_A	-.010 (*.041*)	-.013 (*.040*)	**.116 (*.058*)**	-.069 (*.043*)	-.061 (*.045*)	-.069 (*.037*)	-.024 (*.048*)	**-.144 (*.043*)**	-	-

Bolded parameter estimates are statistically significant at $p < .05$. Depressive symptoms score is square root transformed.

Sleep = met criteria of sleep 8+h or not. Fruits & vegetables = met criteria of 5+ servings or not. Sedentary time = met criteria of <2h sedentary time or not. MVPA = met criteria of 150min MVPA per week or not. Smoking = met criteria of not smoking or not.

b_P = phenotypic association between predictor and outcome. b_A = amount of variance in body mass index attributable to additive genetic influences.

[a] b_P is constrained to be equal for men and women; this model was not performed for smoking as Wald test showed b_p to be statistically significantly different between men and women (Wald = 4.343, p = .037).

Figure A1. Classical univariate twin model for one variable.

The variance of the observed variable (BMI, depressive symptoms score, or number of health behaviors) is decomposed into three components (in circles): additive genetic influences (A), environmental influences that are shared between twin pairs (C), and non-shared environmental influences that are unique to each twin (E). MZ = monozygotic, DZ = dizygotic

Figure A2. Path diagram of a bivariate twin model (only one twin shown for clarity).

The A, C, and E latent variables (in circles) are the additive genetic, shared environmental, and non-shared environmental variance of health behaviors and BMI, respectively. b_P represents the phenotypic effect of health behaviors on BMI, b_A and b_C represent the confounding genetic and shared environmental effect of health behaviors on BMI, respectively. In the phenotypic model (Model 1), b_A and b_C are fixed to zero. In the quasi-causal model (Model 2), b_A and b_C are estimated.
