# Supplementary figures and images for: The pillars of health: influence of multiple lifestyle behaviors on body mass index and depressive symptoms in adult twins
Source: BMC Public Health. 2022 Aug 5;22:1487. doi: 10.1186/s12889-022-13901-7 (PMC9354427; doi:10.1186/s12889-022-13901-7)

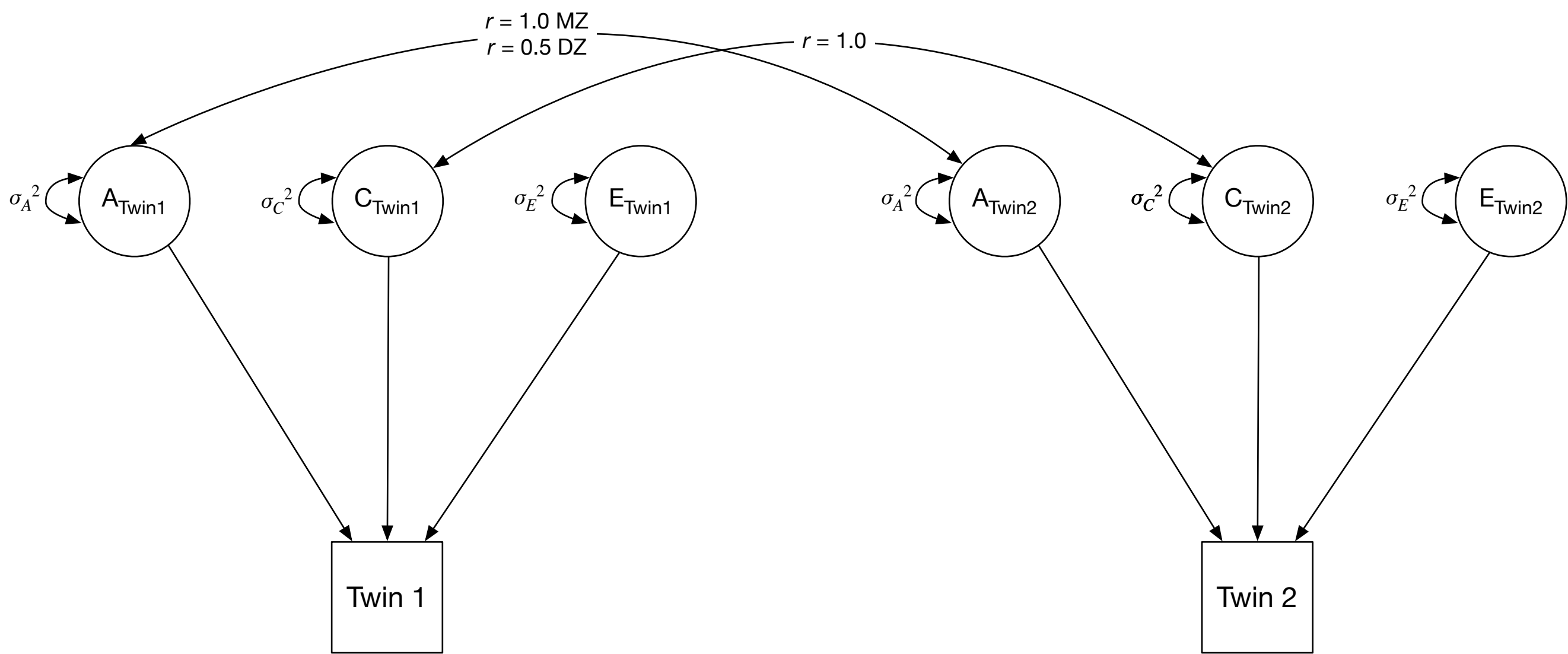

Supplement: Supplementary file 1 — Additional file 1. Appendix Material. [file 12889_2022_13901_MOESM1_ESM.zip › FigureA1-UnivariateTwinModel.pdf]

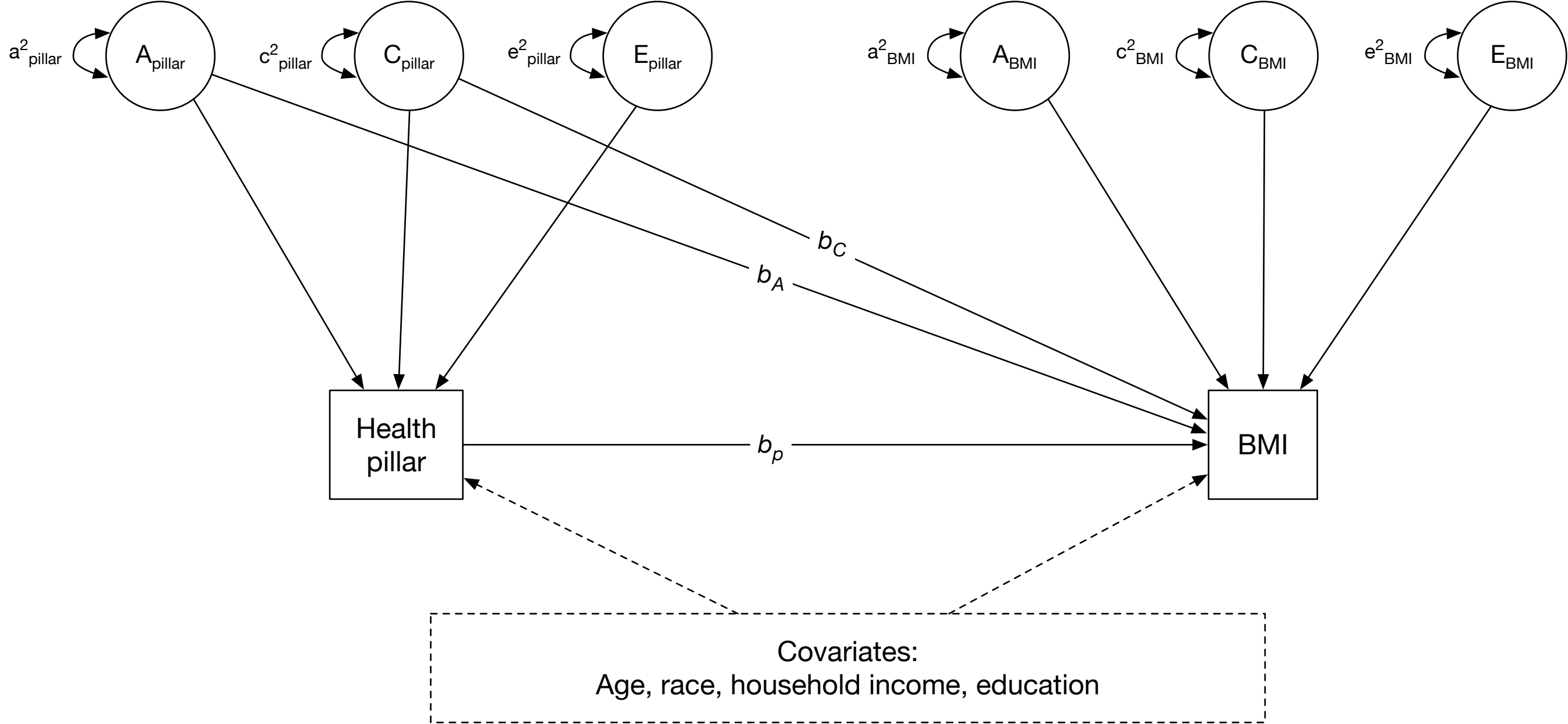

Supplement: Supplementary file 1 — Additional file 1. Appendix Material. [file 12889_2022_13901_MOESM1_ESM.zip › FigureA2-BivariateTwinModel-withcov.pdf]
